# Supplementary material for: Plant leaves inspired sunlight-driven purifier for high-efficiency clean water production
Source: Nat Commun. 2019 Apr 3;10:1512. doi: 10.1038/s41467-019-09535-w (PMC6447597; doi:10.1038/s41467-019-09535-w)
Supplement: Supplementary file 1 — Supplementary Information [file 41467_2019_9535_MOESM1_ESM.pdf]

Supplementary Information

Plant Leaves Inspired Sunlight-driving Purifier for High-efficiency Clean Water Production

Geng et al.

Supplementary Chemicals and Methods

Materials

N-isopropylacrylamide (97%) (NIPAm), N,N'-methylenebis(acrylamide) (99%) (MBAAm), Polyvinyl Alcohol (PVA) (Mw ~1750), ammonium persulfate (APS), N,N,N,N′-tetramethyl-ethylenedianine (TEMED), $MgCl_2$, $CaCl_2$, $CuCl_2$, NaCl, KCl, $CuSO_4$, Na_2SO_4, K_2SO_4, $K_3[Fe(CN)_6]$, sodium dodecylbenzene sulfonate (SDBS), Rhodamine B (RB), Rhodamine 6G (R6G), methyl orange (MO), methyl blue (MB), brilliant yellow (BY), basic fuchsin (BF), methylene blue (MLB), and ethidium bromide (EB) were purchased from Sigma Aldrich and used as received. Ultropure Milli-Q water was used in all experiments.

Estimation of energy conversion.

The energy conversion from solar illumination to thermal transpiration and guttation was calculated by the equation given below:

$$\eta = \mathrm{m}h_{LV}/I \quad (1)$$

where, η is solar thermal conversion efficiency, m is the transpiration and guttation rate, h_{LV} is the total enthalpy of sensible heat (315 J g^{-1}, from *ca.* 25 to 100°C with specific heat of 4.2 J g K^{-1}) and phase change of liquid to water (2256 J g^{-1}), and I is the solar illumination energy.

LCST Determination

The LCST of PN, PNPG, and PNPG-F were determined by a Perkin-Elmer7 series differential scanning calorimeter (model DSC 4, Perkin-Elmer, USA). All samples were allowed to swell in deionized water for at least 24 h at room temperature to reach the equilibrium state. The thermal analysis was performed in a temperature range of 25-45 °C with a heating rate of 3 °C min^{-1} under a dry nitrogen atmosphere with a flow rate of 40 mL min^{-1}.

Deswelling Ratio Measurement

The deswelling rate of the PNIPAm hydrogel under certain irradiation time was determined by the weight of the hydrogel after wiping off the excess water with

moistened filter paper. The weight of the hydrogel was real-time recorded by an analytical balance. Water retention was calculated by the following formula:

$$\text{Water retention} = 100^{(W_t - W_d)}/W_s \tag{2}$$

Where W_t is the weight of the hydrogel at regular time intervals, W_d is the dry weight of the hydrogel and W_s is the weight of the hydrogel balanced in pure water.

Reswelling Kinetics Measurement

The reswelling kinetics of the irradiated hydrogels determined gravimetrically by dipping the samples in deioned water and wiping off the excess water on the surface. The weight was recorded at predetermined dipping time intervals. Water uptake was defined by the following equation:

$$\text{Water uptake} = 100^{(W_t - W_d)}/W_d \tag{3}$$

All the symbols were defined the same as the above.

Characterization.

The porous structure and surface topography of melamine before and after PN growth was observed by using a Sirion-200 scanning electron microscope (FEI, USA). The absorbance spectra of thepurifier were measured using a varian UV-vis spectrophotometer (Cary 5000, USA), coupled with an Agilent integrating sphere. An IR camera (Fluke) were used to measure the temperature increase under solar irradiation. The wettability change of thepurifier after solar irradiation was measured by the contact angle analysis system with a 3.0 µL water droplet. Raman spectra were obtained by using LabRAM HR Evolution (HORIBA Jobin Yvon, France) Raman microscope with a 514 nm laser. Elemental analysis was finished using XPS spectra tanken out by an ESCALAB 250XI photoelectron spectrometer (ThermoFisher Scientific, USA). X-ray diffractions (XRD) were carried out using a D8 Advance X-ray diffractometer with Cu Kα radiation (λ=0.15418 nm, Bruker, Germany). The transpiration and guttation experiment experiments were conducted in the lab using a

solar simulator with an optical filter for the standard AM 1.5 G spectrum. The optical concentration of one sun and two sun is 100 mW cm^{-2} and 200 mW cm^{-2}, respectively. The contact angle was measured using a Dataphysics OCA 15pro CA measuring instrument (DataPhysics Instruments GHPH, Filderstadt).

Supplementary Results and Discussion

Part I: Chemical properties of PNPG-F purifier

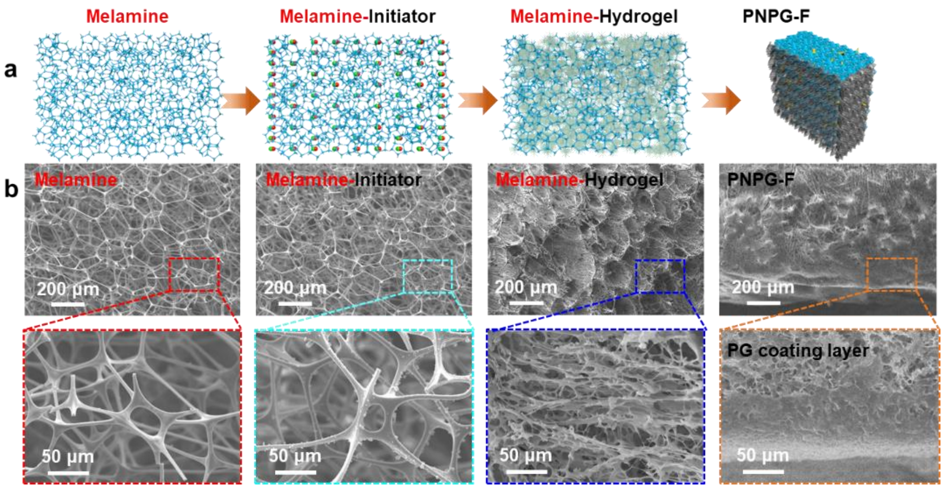

Supplementary Figure 1. Schematic illustration of the preparation of the PNGO-F (a) and the corresponding SEM images (b).

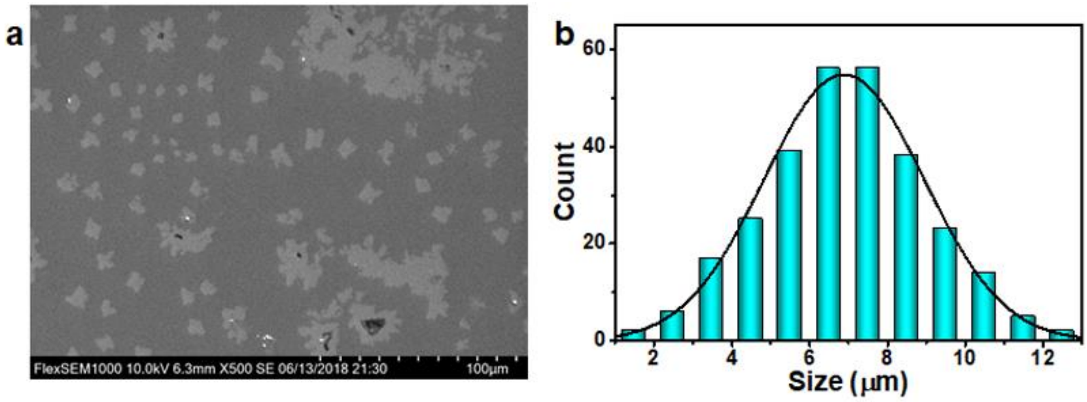

Supplementary Figure 2. SEM images of GO nanosheets (a) and corresponding size distribution (b). GO sheets with an average lateral size of 7.2 μm were synthesized by a modified Hofmann method as we reported previously.[3]

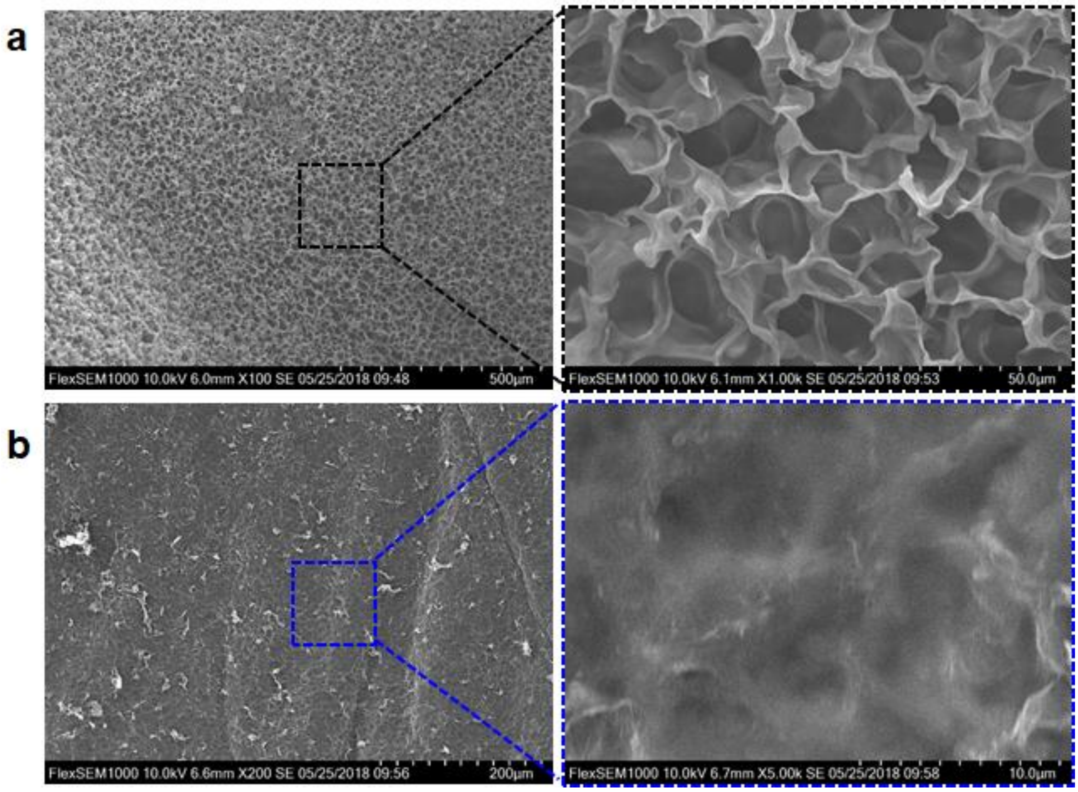

Supplementary Figure 3. SEM images of pure PN hydrogel, a, Crosse-section; b, top-view, and the corresponding magnified images.

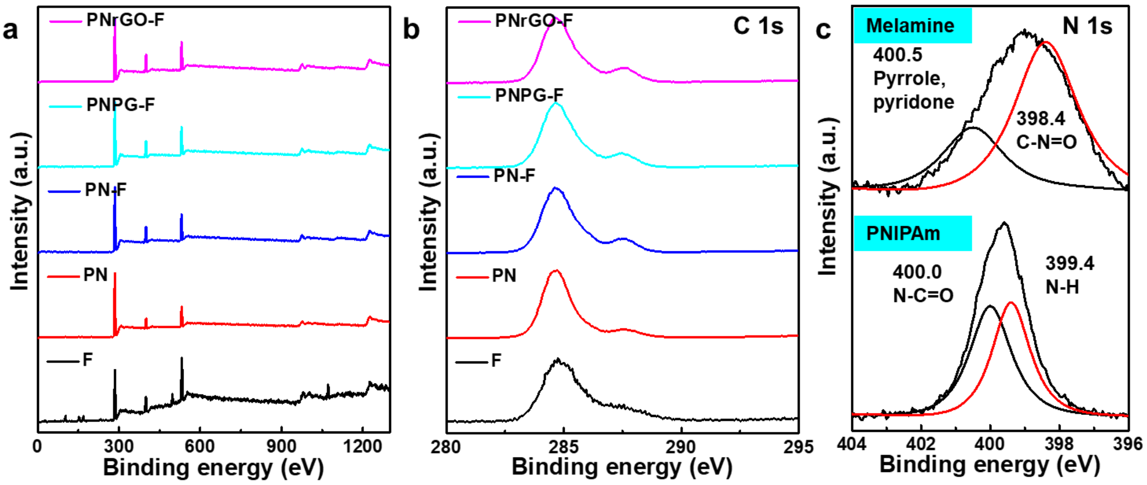

Supplementary Figure 4. a, XPS spectrum. b, C 1s spectrum of various samples. c, N1s spectrum of melamine foam (upper) and PNIPAm (bottom), demonstrating the elemental contents of the melamine foam and the successful modification of PNIPAm on the surface of GO nanosheets, respectively.

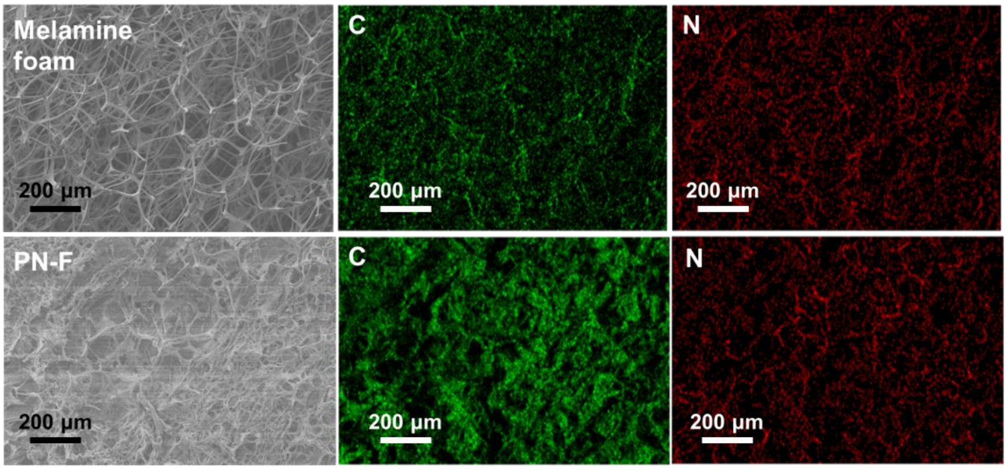

Supplementary Figure 5. Elemental mapping image of Melamine foam and PN-F.

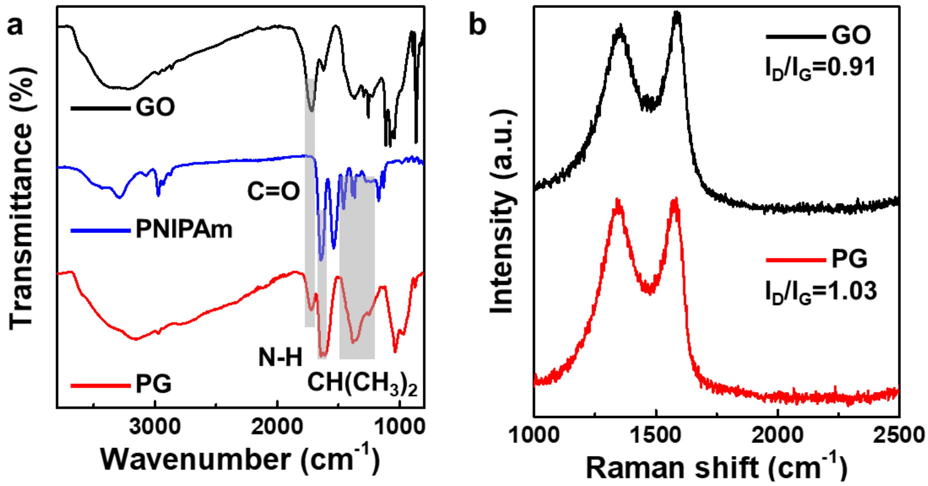

Supplementary Figure 6. ATR-FTIR spectra of GO, PNIPAm and PG. b, Raman characterizations of GO and P-GO.

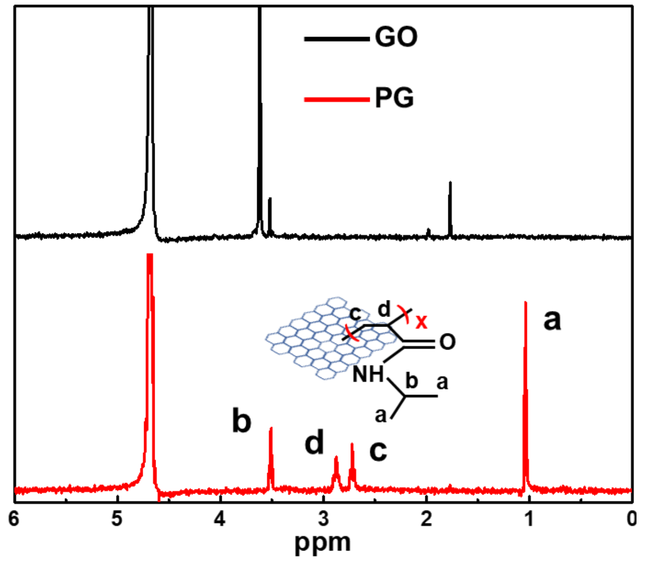

Supplementary Figure 7. 1H NMR spectra of GO and PG in D_2O. The 1H NMR spectroscopic study of the PG demonstrated the successful modification. After the PNIPAM was grafted on the surface GO sheets, the proton peak of CH_3 (a), CH (b), CH_2 (c) and CH (d) and groups appear.

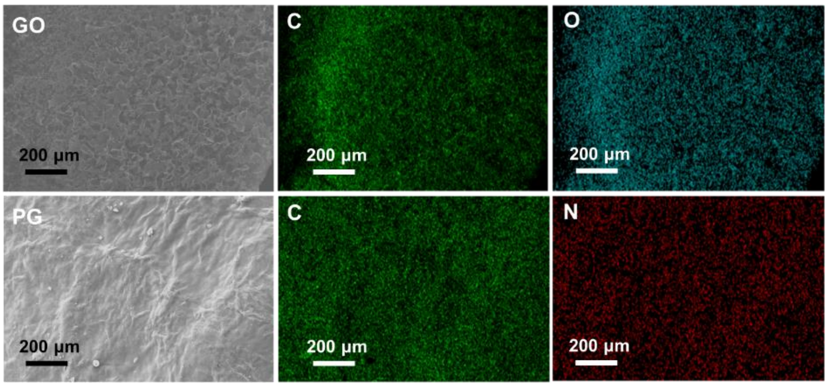

Supplementary Figure 8. Elemental mapping image of GO and PG.

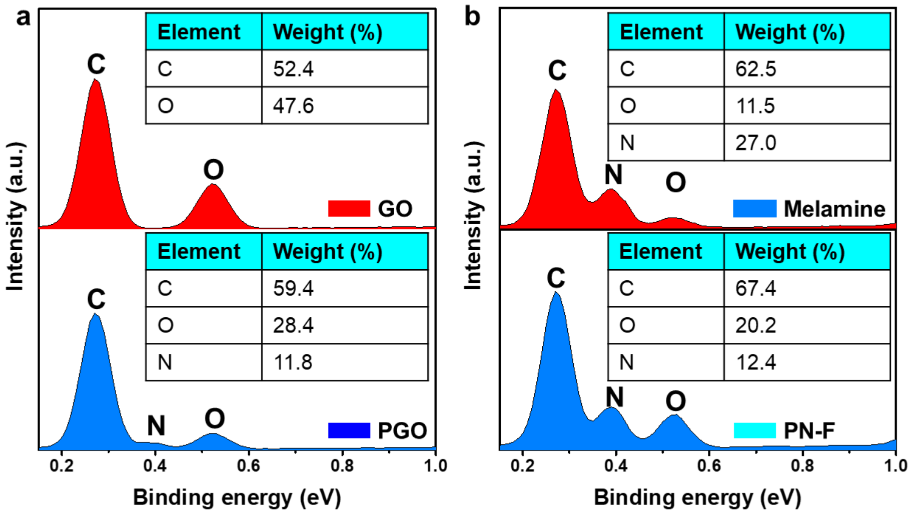

Supplementary Figure 9. EDS survey of GO (a), PG (a), Melamine foam (b) and PN-F (b) respectively.

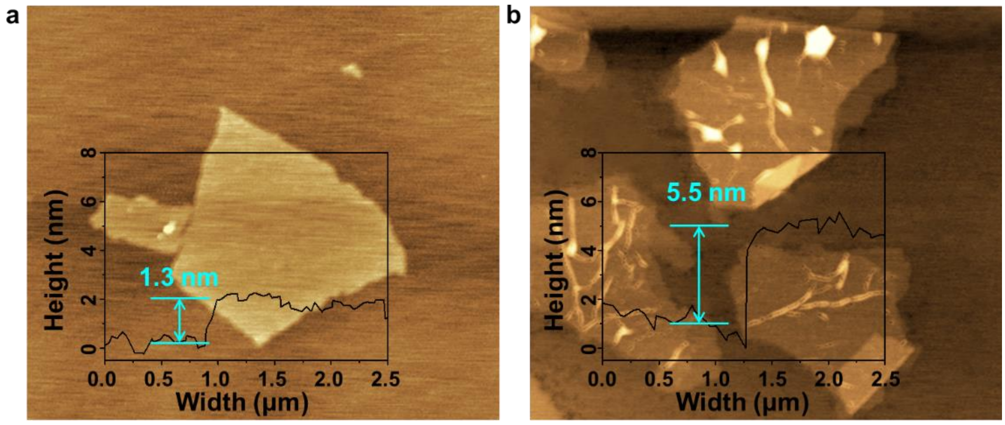

Supplementary Figure 10. An AFM image of GO and PG on the surface of mica. The height profile demonstrates that the successful modification of PNIPAm.

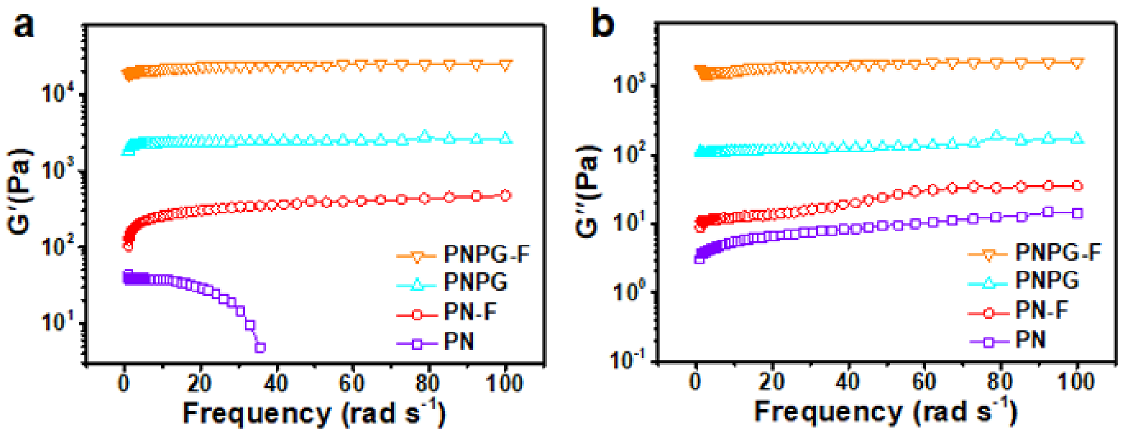

Supplementary Figure 11. Dynamic mechanical analysis of the storage modulus (a, G′) and loss modulus (b, G″) of various samples.

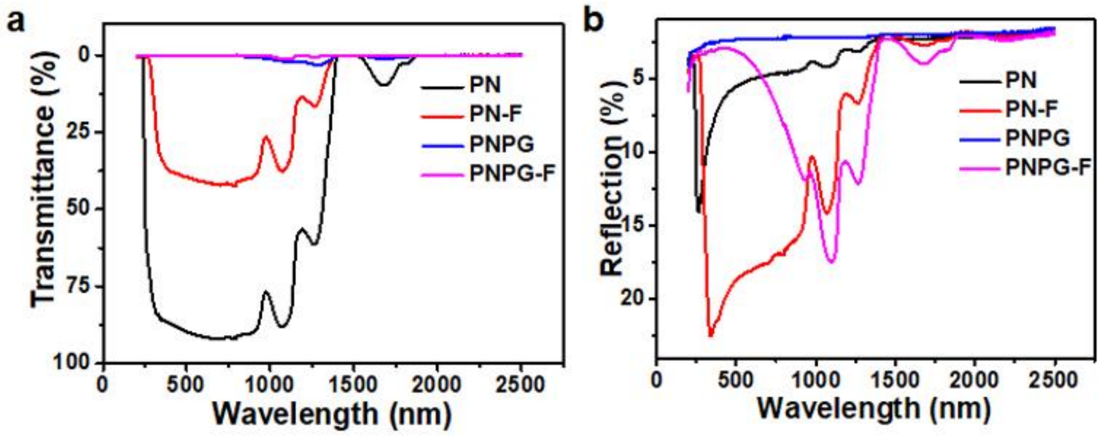

Supplementary Figure 12. a, UV-vis NIR transmittance spectra of samples with size of 2.0×2.0×0.5 cm. b, UV-vis NIR reflection spectra of various samples in 5 mm thick.

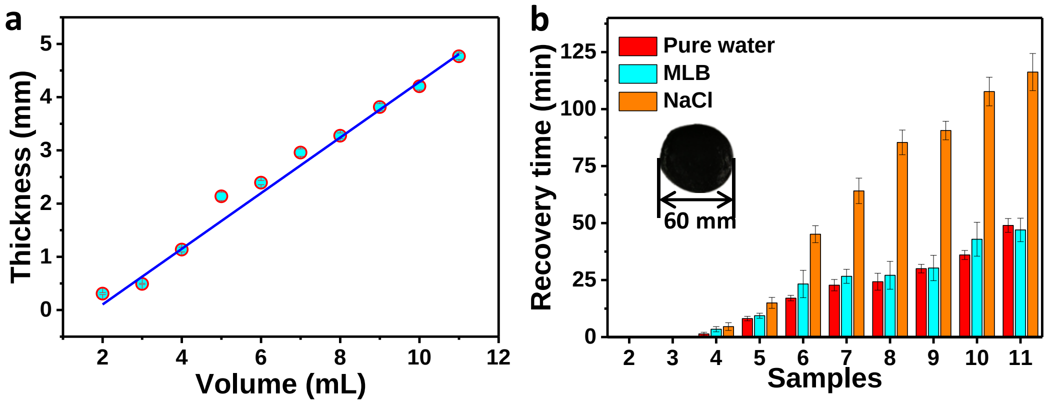

Supplementary Figure 13. a, Method to control the thickness of the hydrogel: various volume of NIPAm solution were added into a petri dish with 60 mm in diameter. After gelation at room temperature, circular hydrogel with various thickness was obtained. b, Reversion time of pure PNGO hydrogel with various thickness in pure water, NaCl aqueous solution (3.5wt%) and in MLB aqueous solution (200 mg L^{-1}) corresponding to different volumes in Fig. S8(a). Error bars were determined based on the standard deviation of three samples. Source data are provided as a Source Data file.

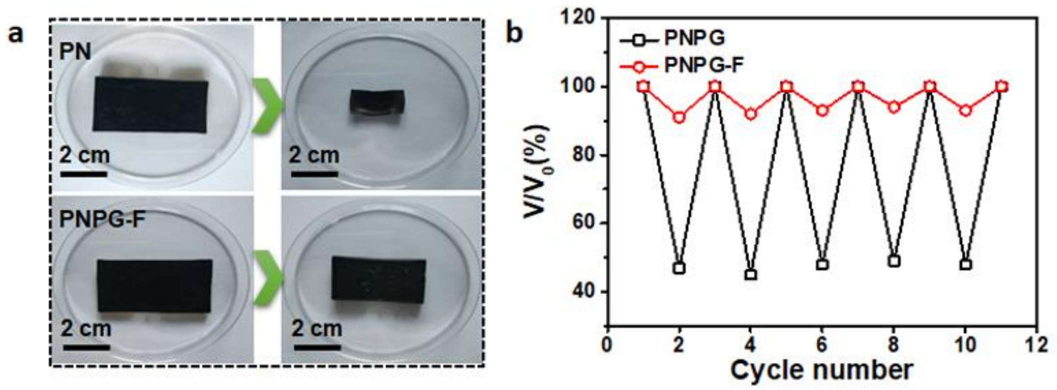

Supplementary Figure 14. a, Photos show collapse of a cuboid PNPG-F and PNPG (2.0×2.0×0.5 cm) being irradiated under sunlight for 10 mins to remove adsorbed water. b, Volume cyclicity of PNPG and PNPG-F.

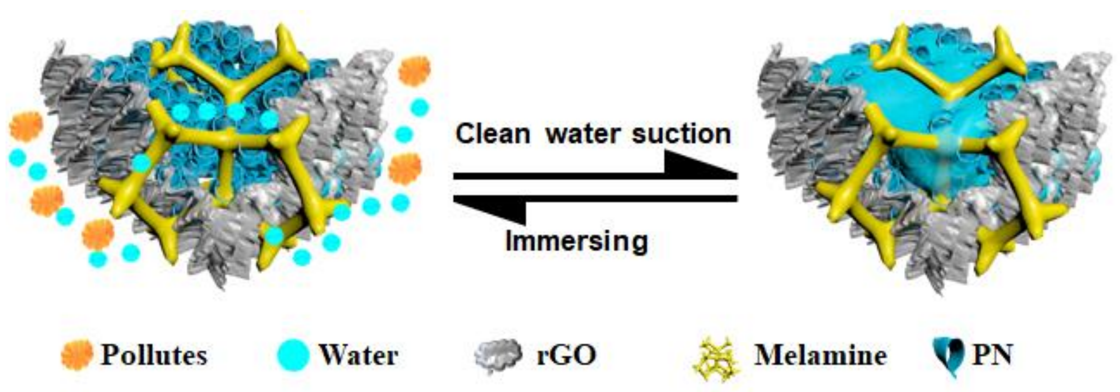

Supplementary Figure 15. Schematic of high-efficiency water suction and pollute rejection.

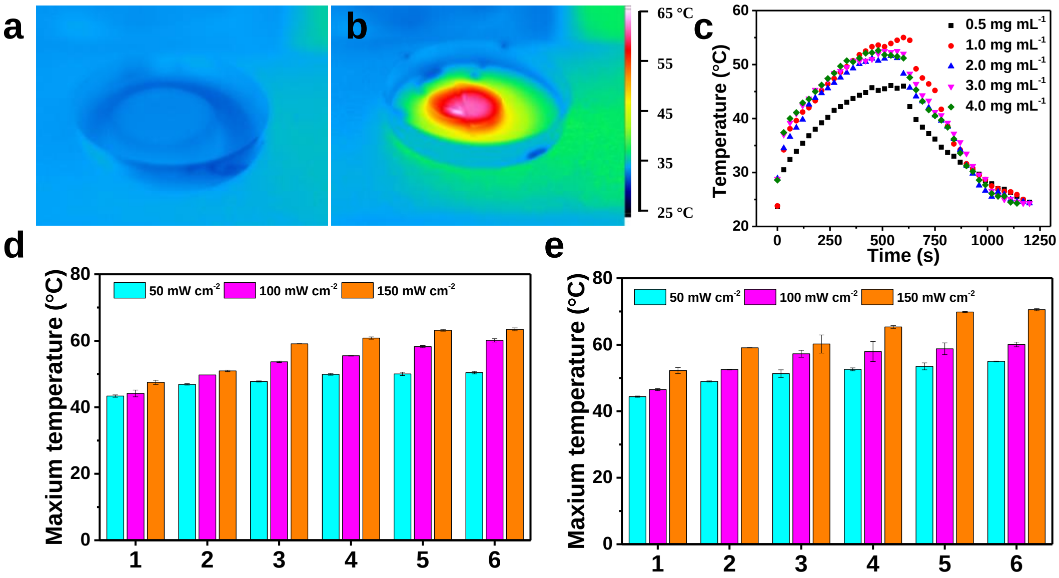

Supplementary Figure 16. a and b, Digital photos of wet hydrogel without (a) and with PG (b). c, Temperature variation of PNPG-F with various oxidized graphene concentration over time. The ambient temperature is ~ 22 °C, and the results obtained with utilization of wet hydrogel. d and e, The maximum temperature of PNPG with different PG concentration (d, unit is mg mL^{-1}) and rGO concentration (e, unit is mg mL^{-1}) under increasing optical concentration. Error bars reflect the standard deviation of three samples. Source data are provided as a Source Data file.

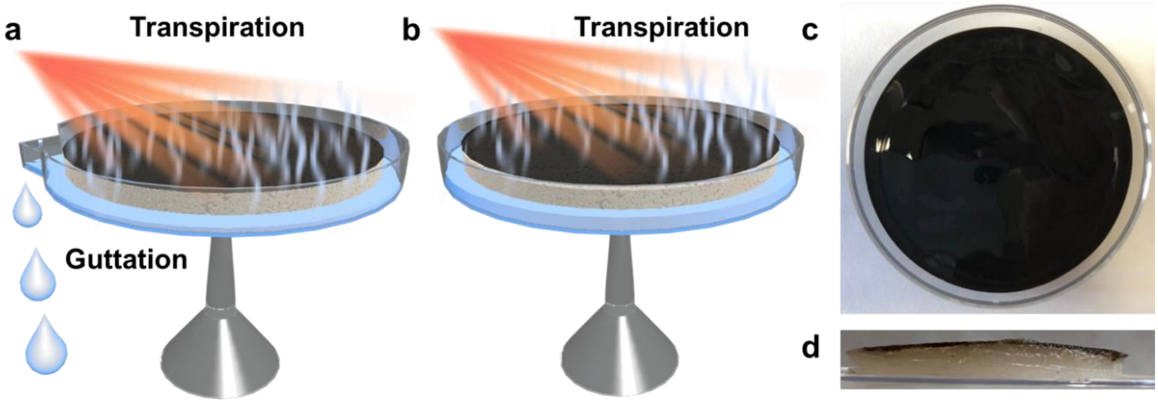

Supplementary Figure 17. Schematic illustration of the method to calculate the water generation rate. a, transpiration and guttation; b transpiration; c and d, Digital images of PNGO-F c (top-view), d (side-view). Only upper surface was coated with PG to avoid the heating of the size. The surface of the upper surface was much larger than the size of sunlight, thus the size change of the samples as water release can be avoided.

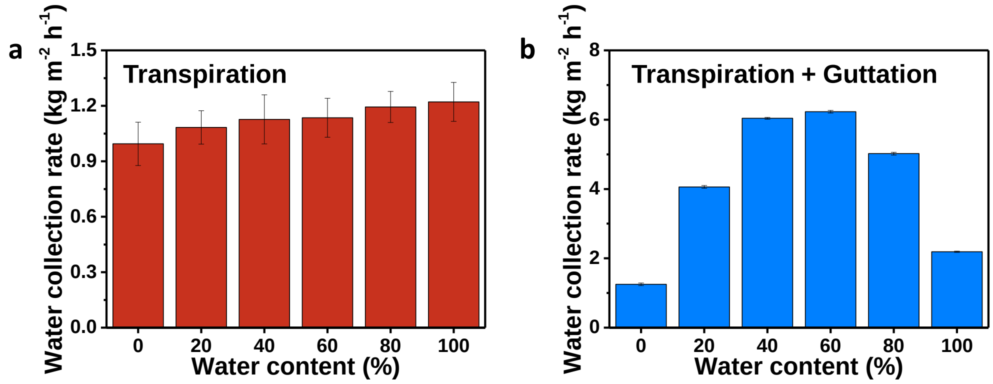

Supplementary Figure 18. The effectiveness of water content on the rate of water collection. Error bars were determined based on the standard deviation of three samples for three times. Source data are provided as a Source Data file.

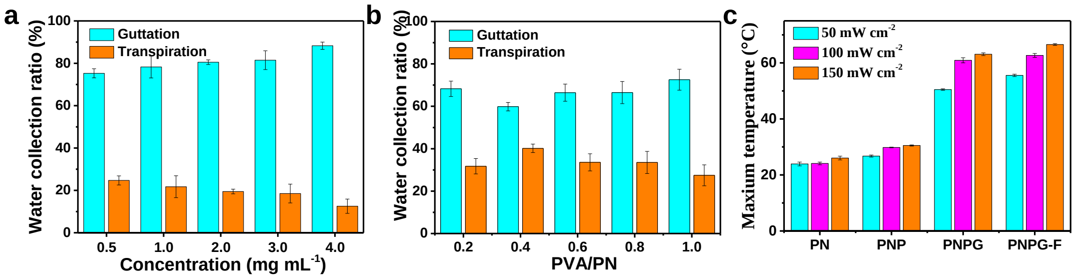

Supplementary Figure 19. a, The mass loss ratio of water via evaporation for a single PNPG-F purifier with various PG concentration. The weight of the hydrogels is ~6 g. The sizes of the hydrogels used here are 2×2×0.5 cm. b, The mass loss ratio of water via evaporation and compression for a single PNPG-F hydrogel with various weight ratio of PVA and PN. The weight of the hydrogels is ~6 g. The sizes of the hydrogels used here are 2×2×0.5 cm. c, Water release of samples under one sun irradiation after being immersed in pure water. Error bars were determined based on the standard deviation measured for three times. Source data are provided as a Source Data file.

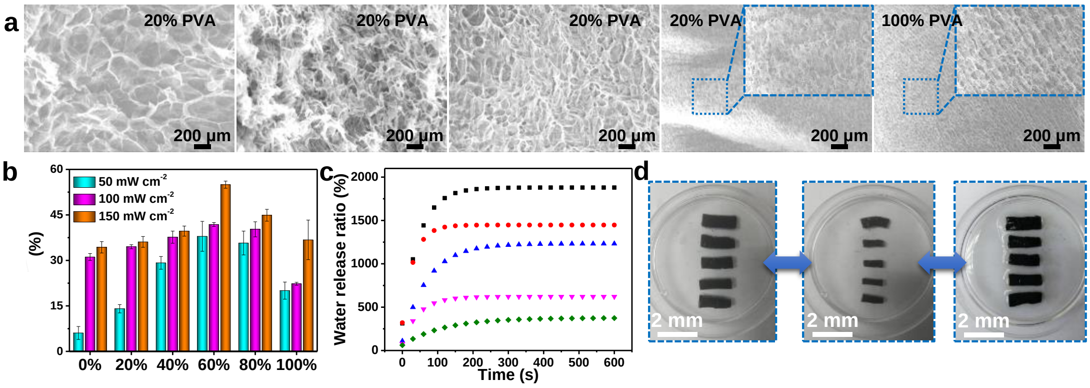

Supplementary Figure 20. a, SEM images of PNPG-F with various PVA contents. The ratio of PVA was determined by the amount of PN. The concentration of oxidized graphene used here is 5.0 mg mL^{-1} b, Water release ratio of PNPG-F with various PVA contents under one sun irradiation. c, Water release ratio of PNPG-F under one sun irradiation as a function of time. d, Rapid deformation of a PNPG hydrogel. Pictures of the hydrogel rod (original shape; 2 mm in length, 0.2 mm in width and 0.2 mm in height). Photos show changes in length under one sun irradiation (from left to right). Error bars were determined based on the standard deviation measured for three times. Source data are provided as a Source Data file.

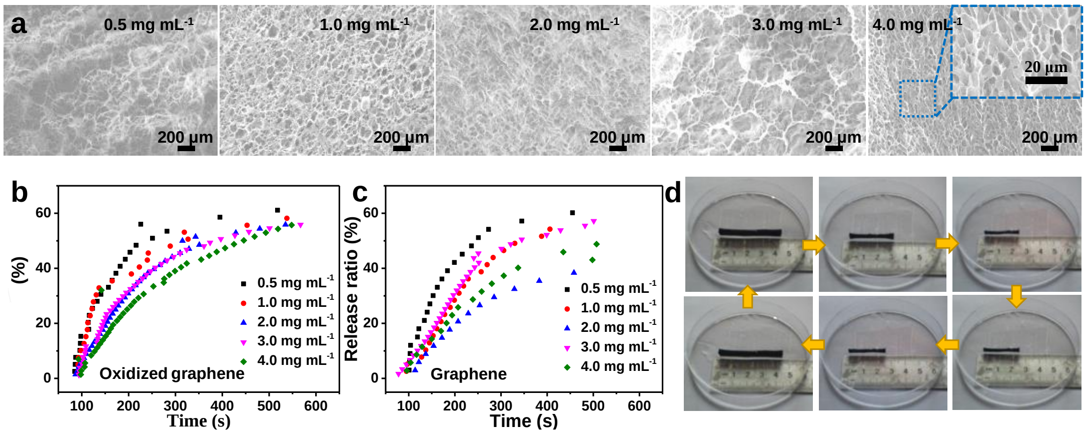

Supplementary Figure 21. a, SEM images of PN hydrogel with various GO concentration. b, Water release ratio of PNPG-F purifier under illumination of one sun as a function of time. c, Water release ratio of PNrGO under illumination of one sun as a function of time. d, Rapid deformation of a PNGO hydrogel rod. Pictures of the hydrogel rod (original shape; 4 cm in length and 0.5 mm in diameter). Photos showing changes in length under one sun irradiation (top line) and without irradiation (bottom line).

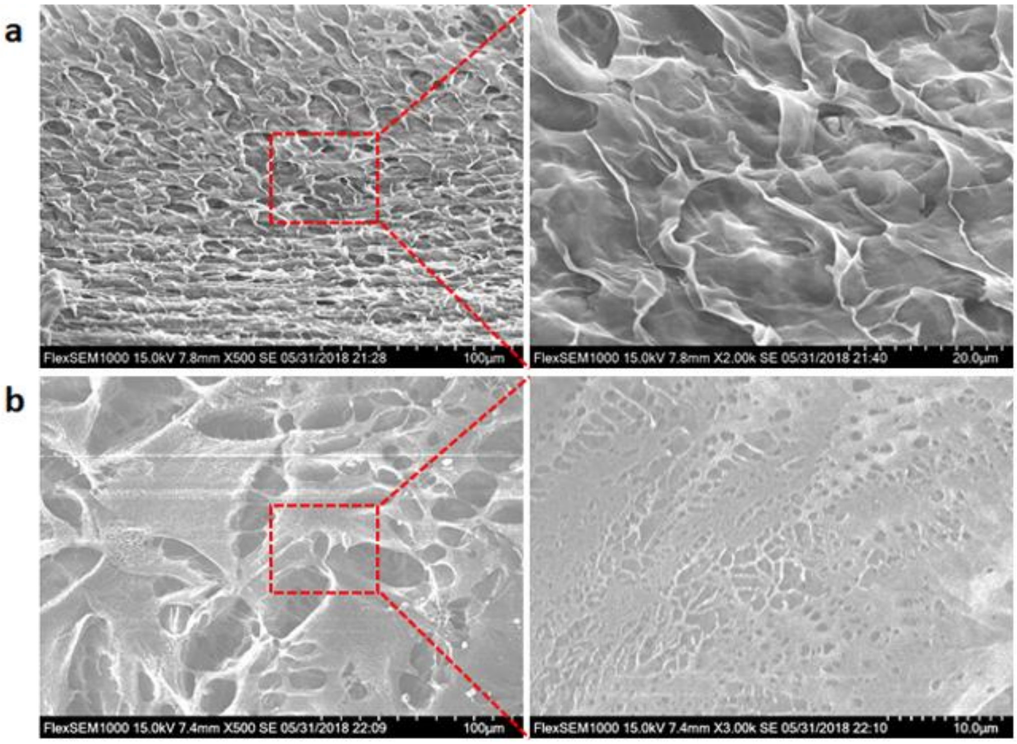

Supplementary Figure 22. SEM images of PN hydrogel without (a) and with PVA (b) (weight ratio of PN and PVA is 0.5).

Part II: Theoretical analysis and the discussion of the mechanism
Simulation details.
The OPLS-AA + SPC/E force fields[4,5] were used in MD simulations which have been performed by using LAMMPS.[6] The initial molecular configuration was constructed as follows: In the first step of the structure generation, a monomer of the NIPAM molecule was generated and placed in a simulation cell. The adjacent two monomers were placed with a bond-distance of backbone atoms, which was repeated for obtaining a single 30-mer oligomers chain (PNIPAm). We constructed the atomistic model of 16 PNIPAm chains and 7680 water molecules (SPC/E model) in a cubic simulation cell with periodic boundary and lattice parameters of a=57.1 Å, b=60.9 Å, c=89.5 Å, which corresponds to a PNIPAm density of 217.33 $Å^2$/chain closing to our experiments. The energy of the resulting system was minimized using the steepest descent and conjugate gradient algorithms. After minimization, we performed system equilibration in the NPT ensemble at 290K and 1 bar of pressure, employing the Nose-Hoover thermostat and barostat for at least 10 ns. The polymer backbone was firstly extended and kept rigid during the energy minimization and subsequent NPT run. After the equilibration stage, we constrained the movement of one bottom backbone atom of each chain and relaxed other atoms for simulating the anchored PNIPAm chains on melamine skeleton (PN-F system) as one PNIPAM chain is shown in supplementary Fig. S13. Both simulations of anchored and free chains (PN system) were continued for 100 ns in the NVT ensemble at temperatures at 280 K, and then they were further relaxed at temperature heating to 340 K for another 100 ns. This simulation time was enough to make sure that the stretched or the collapsed conformation were reached.[6] The Lennard-Jones (LJ) and point charge parameters of the PNIPAM force fields were used after the previous work.[5,6] Also, the geometric mean mixing rule was applied for describing intramolecular and intermolecular interactions. All interactions were controlled within

a cutoff radius of rc = 1.2 nm. For the long-ranged electrostatic and van der Waals interactions, the particle-particle particle-mesh (PPPM) method was used. The SHAKE algorithm was used to constrain the bond lengths and the angles of water molecules. All simulations were performed using a time step of 2 fs.

Hydrogen bond analysis.

Hydrogen bond was crucial to determine LCST behavior,[8-11] which also indicated the varied hydrophilicity of PNIPAm. To investigate the amide group interactions with water in further detail, we examined the number of intermolecular hydrogen bonds between water and hydrophilic group of polymer (−N−H…H_2O and −C=O…H_2O). And the hydrogen bonds between polar groups and water molecules were measured using the geometric criteria:[9] $R_{OO} \leq 3.6$, $R_{OH} \leq 2.45$, $\phi \leq 30°$. Here, R_{OO} represents the distance between acceptor atom and donator atom, while ROH is the distance between donator atom and hydrogen atom. Specifically, two types of hydrogen bonds between the polar groups (−C=O and −N−H of PNIPAMs)) and water molecules were examined: the contribution between $O_{(PNIPAMs)}$ acceptor and $OH_{(water)}$ donator, and the contribution between $NH_{(PNIPAMs)}$ donator and $O_{(water)}$. The angle ϕ is the angle between acceptor atom, donator atom and hydrogen atom of donator.

Total Surface Area between PNIPAm chains and water molecules.

To analyse contact interfaces between PNIPAm chains and water, we monitored the variations in the total surface area of the PNIPAm chains and amide group which are accessible to the water molecules.

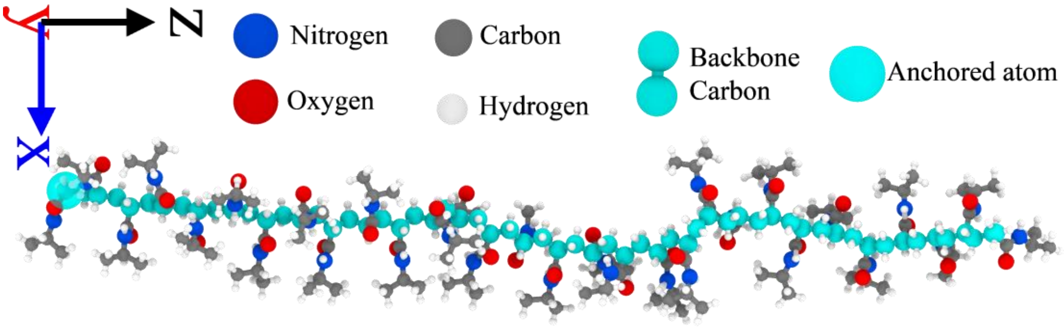

Supplementary Figure 23. A model of 30 monomer chain, in which a backbone carbon atom acted as an anchored atom.

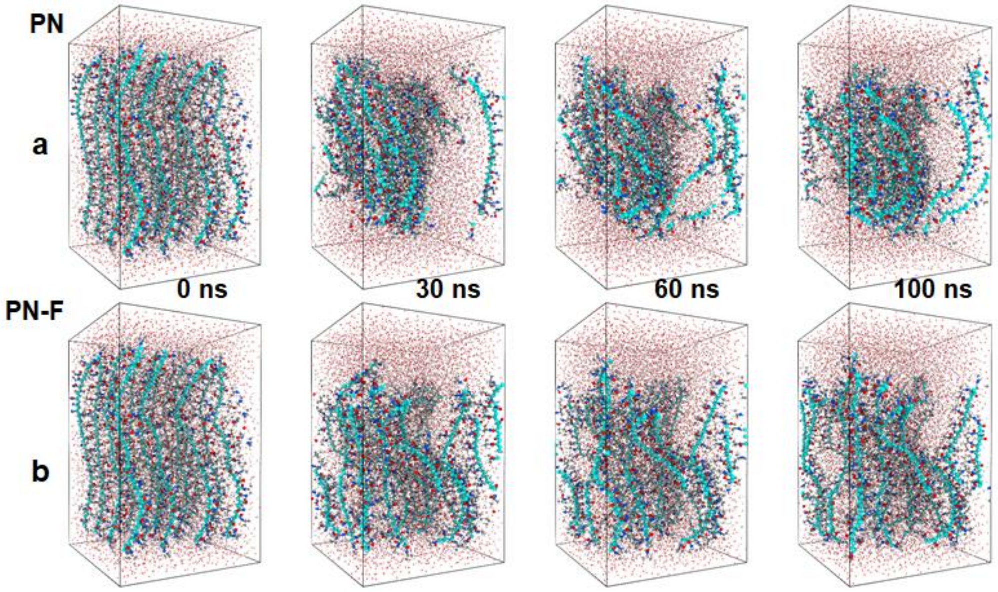

Supplementary Figure 24. Snapshots of PN systems at 280 K, hydrogen bonds and density profiles of PN-F and PN systems. a, b, Perspective view of snapshots after relaxed initial structure of PN (free chains) and PN-F (anchored one ending atom of the backbone in each chain) systems at 280K. c, d, Density profiles exhibit the inhomogeneous density distribution of C atoms in amide group and O atoms in water.

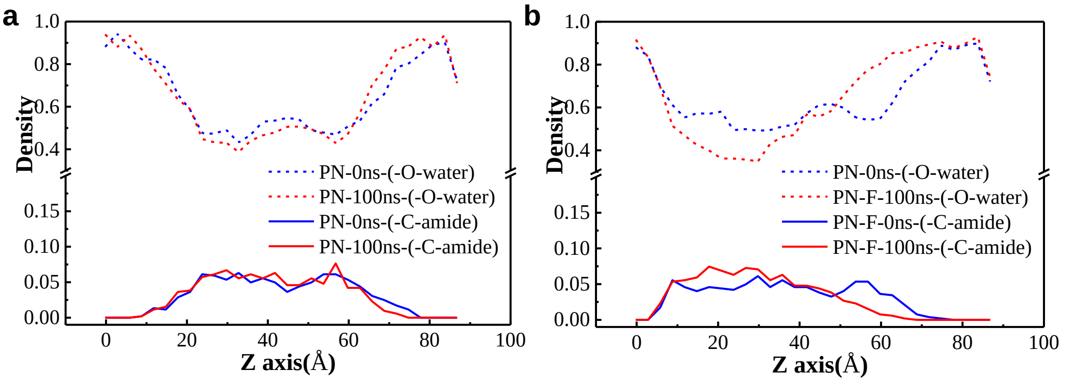

Supplementary Figure 25. a, b, Density profiles exhibit the inhomogeneous density distribution of C atoms in amide group and O atoms in water during heating at temperature of 340K. Source data are provided as a Source Data file.

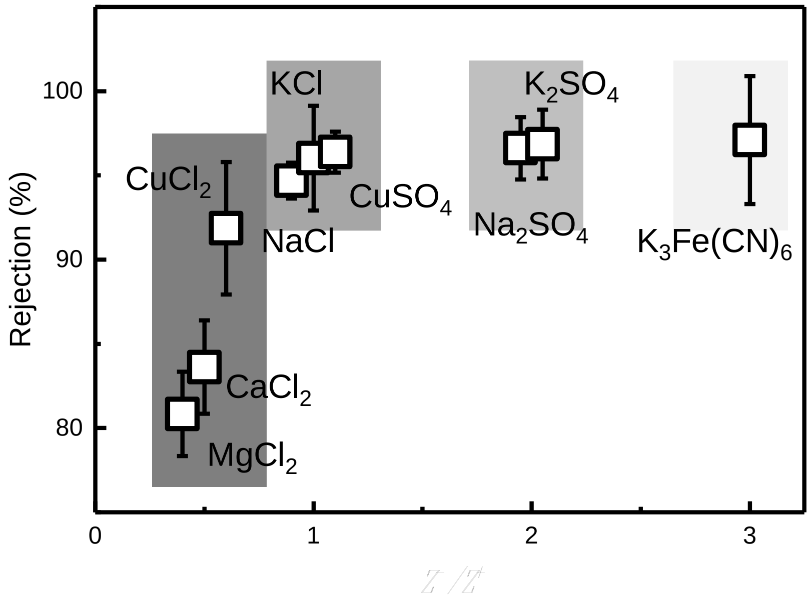

Supplementary Figure 26. Rejection of electrolyte salts with respect to the ion valence ratio when concentration is 0.1 mol L^{-1}. The position of Z^-/Z^+ is shifted to left or right for visualization.

Dye molecule	MW (gmol^{-1})	Size (Å)	Charge	c_1 (X mol L^{-1})	c_2 (X mol L^{-1})	c_3 (X mol L^{-1})
Rhodamine B, RB	479.01	16.82×12.97	+	89.40	92.03	93.36
Rhodamine 6G, R6G	479.02	16.19×12.27	+	92.03	93.73	93.99
Methylene blue, MLB	319.85	14.62×7.17	+	93.36	95.92	96.18
Basic fuchsin, BF	337.86	11.12×12.28	+	93.11	96.19	96.26
Methyl blue, MB	799.80	18.10×12.17	-	93.73	96.73	96.89
Methyl orange, MO	327.33	16.99×5.53	-	93.99	97.16	98.33
Evans blue, EB	960.80	27.77×10.02	-	95.52	99.41	99.55
Brilliant yellow, BY	624.55	27.49×11.12	-	95.92	99.98	99.90

Supplementary Table 1. Separation performance of the PNGO membrane for different dye molecules.

Part III: Feasible demonstration of the as-prepared purifier

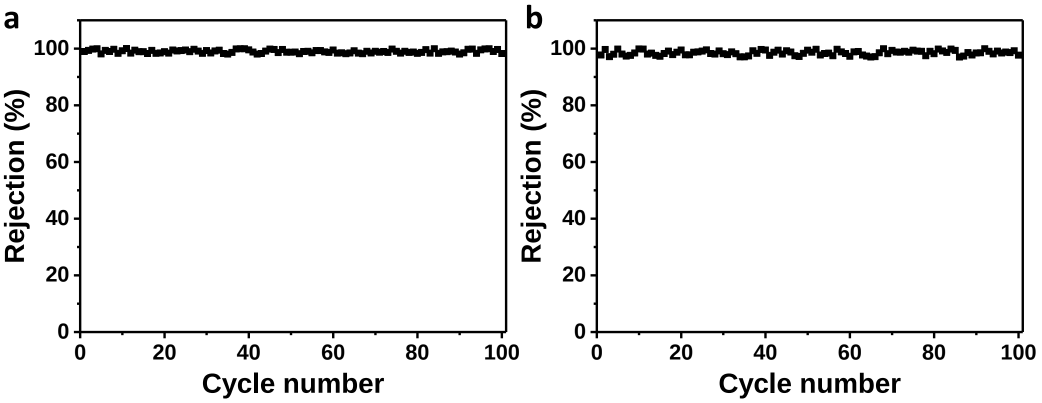

Supplementary Figure 27. Cyclability of rejection of PNGO-F for separation of $K_3[Fe(CN)_6]$ (a) and RB (b).

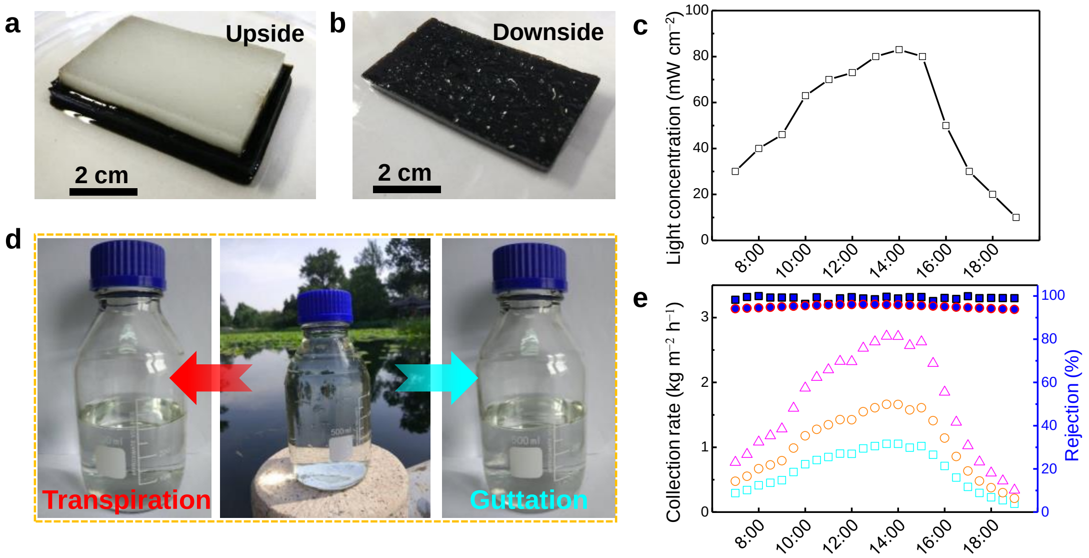

Supplementary Figure 28. Outdoor solar water collection using PNPG-F in natural sunlight. a and b, Photographs of a large-scale PNPG-F purifier. c, Solar radiation recorded over time on a sunny day from 07:00 to 19:00. d, Photographs of purified water via transpiration and guttation from Tsinghua University. e, The amount of purified water during 12 h of outdoor solar desalination of sample from the Bohai Sea.

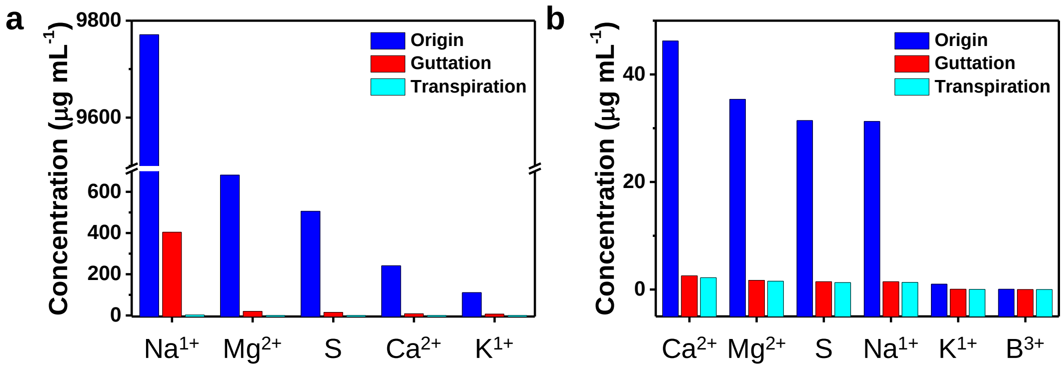

Supplementary Figure 29. The concentrations of ions in the actual sea and lake water by PNGO-F motor under sunlight irradiation.

Supplementary Reference

1. Wu, M. M. *et al.* Chemical approach to ultrastiff, strong, and environmentally stable graphene films. *ACS Appl. Mater. Interfaces* **10**, 5812-5818 (2018).
2. Ma, C. B. Shi, Y. Pena, D. A. Peng, L. L. & Yu, G. H. Thermally responsive hydrogel blends: a general drug carrier model for controlled drug release. *Angew. Chem. Int. Ed.* **127,** 7484-7488 (2015).
3. Jorgensen, W. L. Maxwell, D. S. & Tirado, R. J. Development and testing of the OPLS all-atom force field on conformational energetics and properties of organic liquids. *J. Am. Chem. Soc.* **118,** 11225-11236 (1996).
4. Berendsen, H. J. C. Grigera, J. R. & Straatsma, T. P. The missing term in effective pair potentials. *J. Phys. Chem.* **91,** 6269-6271 (1987).
5. Plimpton, S. J. Fast parallel algorithms for short-range molecular dynamics. *J. Comput. Phys.* **117,** 1−19 (1995).
6. Walter, J. Ermatchkov, V. Vrabec, J. & Hasse, H. Molecular dynamics and experimental study of conformation change of poly (N-isopropylacrylamide) hydrogels in water. *Fluid Phase Equilibr.* **296,** 164-172 (2010).
7. Boţan, V. Ustach, V. Faller, R. & Leonhard, K. Direct phase equilibrium simulations of NIPAM oligomers in water. *J. Phys. Chem. B* **120,** 3434-3440 (2016).
8. Deshmukh, S. A. Sankaranarayanan, S. K. R. S. Suthar, K. & Mancini, D. C. Role of solvation dynamics and local ordering of water in inducing conformational transitions in poly (N-isopropylacrylamide) oligomers through the LCST. *J. Phys. Chem. B* **116,** 2651–2663 (2012).
9. de Oliveira, T. E. Marques, C. M. & Netz, P. A. Molecular dynamics study of the LCST transition in aqueous poly (Nn-propylacrylamide). *Phys. Chem. Chem. Phys.* **20,** 10100-10107 (2018).
10. Pelton, R. Poly (N-isopropylacrylamide)(PNIPAM) is never hydrophobic. *J. Colloid Interface sci.* **348,** 673-674 (2010).

11. Luzar, A. & Chandler, D. Structure and hydrogen bond dynamics of water–dimethyl sulfoxide mixtures by computer simulations. *J. Chem. Phys*. **98,** 8160-8173 (1993).
