# Supplementary material for: Plant leaves inspired sunlight-driven purifier for high-efficiency clean water production
Source: Nat Commun. 2019 Apr 3;10:1512. doi: 10.1038/s41467-019-09535-w (PMC6447597; doi:10.1038/s41467-019-09535-w)
Supplement: Supplementary file 3 — Description of Additional Supplementary Files [file 41467_2019_9535_MOESM3_ESM.docx]

**Description of Supplementary Files**

**File Name:** Supplementary Movie 1

**Description:** Volume recovery monitoring of dried PNPG.

**File Name:** Supplementary Movie 2

**Description:** Volume recovery monitoring of dried PNPG-F.

**File Name:** Supplementary Movie 3

**Description:** Volume recovery monitoring of dried PNrGO-F.

**File Name:** Supplementary Movie 4

**Description:** Real-time measurement of the temperature increasing of PN and PNPG under one sun irradiation.

**File Name:** Supplementary Movie 5

**Description:** Water collection from transpiration and guttation of a single PNPG-F purifier under one sun irradiation.
